# A new treatment for endoscopic ultrasound-guided vascular intervention: coiling with sclerotherapy for esophageal varices

**DOI:** 10.1055/a-2443-3851

**Published:** 2024-11-08

**Authors:** Kazunori Nagashima, Manabu Ishikawa, Yasunori Inaba, Ken Kashima, Yasuhito Kunogi, Fumi Sakuma, Atsushi Irisawa

**Affiliations:** 1 12756 Gastroenterology, Dokkyo Medical University, Shimotsuga, Japan

In recent years, interventional endoscopic ultrasound (EUS) has been applied to the treatment of vascular lesions such as isolated gastric varices and intractable gastrointestinal bleeding 1 . For esophageal varices, it is usual to perform endoscopic injection sclerotherapy (EIS) or endoscopic variceal ligation (EVL) 2 3 ; however, varices of thick diameter without palisade vessels (so-called pipeline varices) are often difficult to treat 4 . This report is the worldʼs first of a new treatment for EUS-guided vascular intervention using a combination of coiling with sclerotherapy for esophageal varices.

Video 1 shows a typical case. The patient, a 57-year-old man, had alcoholic cirrhosis and thick esophageal varices ( Fig. 1 ). Contrast-enhanced computed tomography (3D-CT) and an EUS showed pipeline varix hemodynamics that fed from the left gastric vein to the azygos vein ( Fig. 2 and Fig. 3 ). First, an overtube was inserted; EVL was then performed on the varices on the proximal side. The varices were then punctured using a 19G fine-needle aspiration needle (EZ shot3 plus; Olympus Corp., Tokyo, Japan) near the junction. A 0.035-inch hydrocoil (Azur; Terumo Corp., Tokyo, Japan) was placed. The blood flow was checked by injecting a contrast medium and using the EUS color Doppler function and some additional coils were placed. A sclerosant (ethanolamine oleate) was injected into the feeder vessel ( Fig. 4 ), with subsequent cessation of the blood flow. After 1 week, it was confirmed that blood flow had been completely stopped with only the one session of treatment ( Fig. 5 ). Moreover, no adverse events occurred.

A new single-session treatment is performed for pipeline esophageal varices consisting of endoscopic ultrasound-guided vascular intervention with a combination of coiling and sclerotherapy.Video 1

**Fig. 1:**
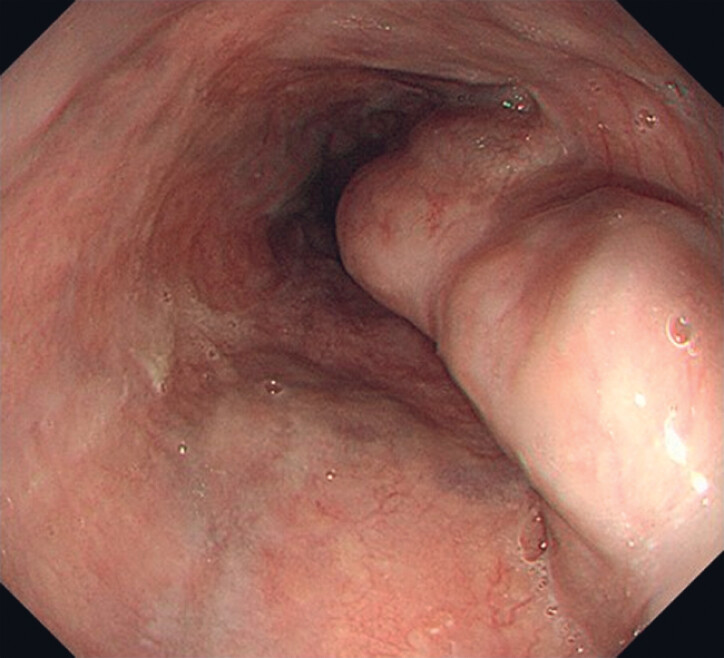
Endoscopic image of thick, highly developed varices at 2 oʼclock.

**Fig. 2:**
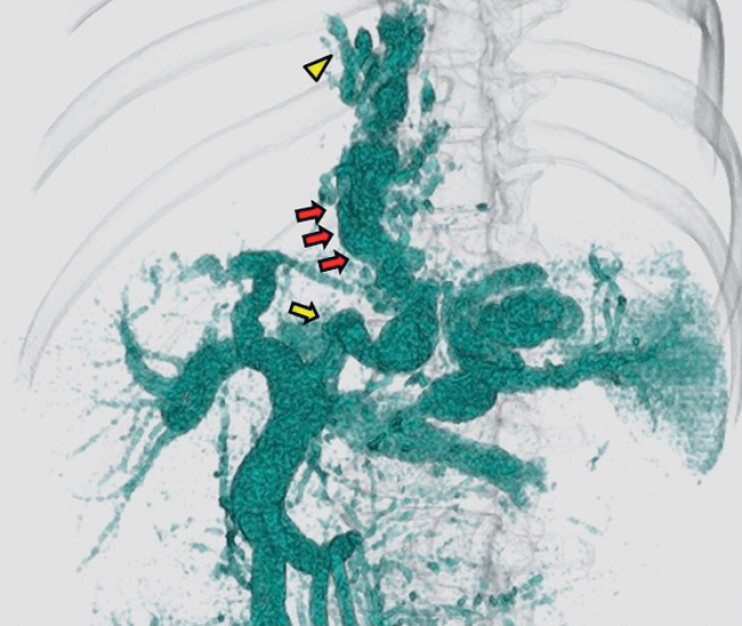
3D computed tomography image showing the hemodynamics of a pipeline varix (red arrows) that fed from the left gastric vein (yellow arrow) to the azygos vein (yellow arrowhead).

**Fig. 3:**
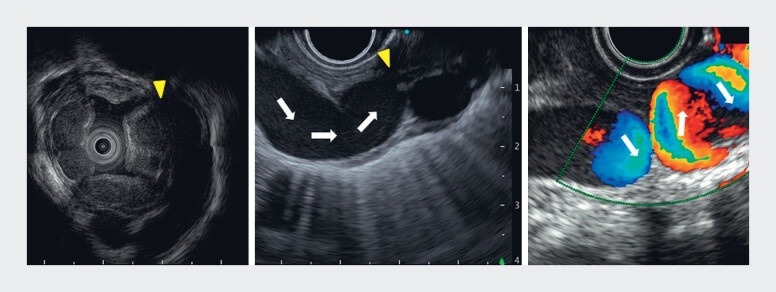
Endoscopic ultrasound images showing varices in which there were no palisade vessels (yellow arrowheads), with the pipeline varix flowing from left to right (white arrows).

**Fig. 4:**
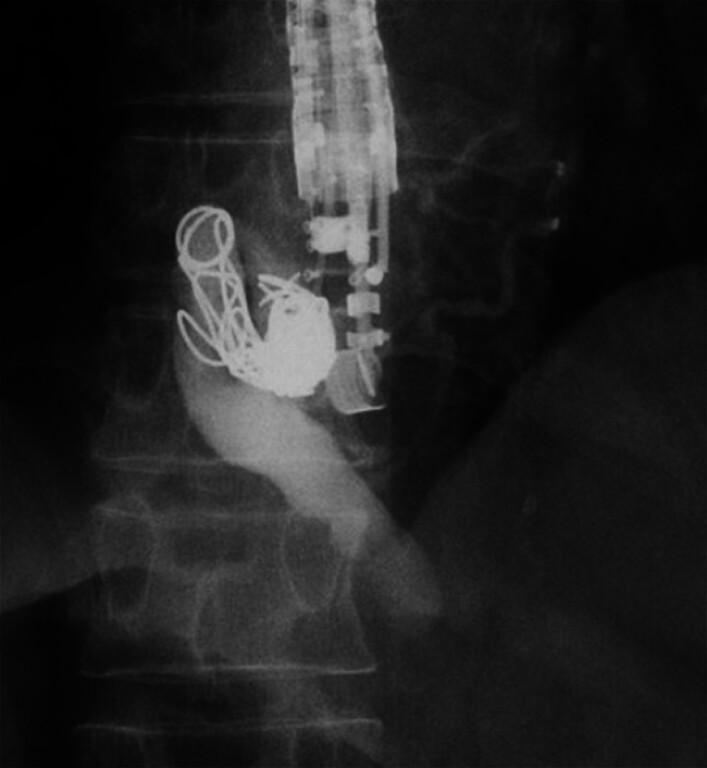
Fluoroscopic image showing additional coils that were placed, with injection of sclerosant into the feeder vessel.

**Fig. 5:**
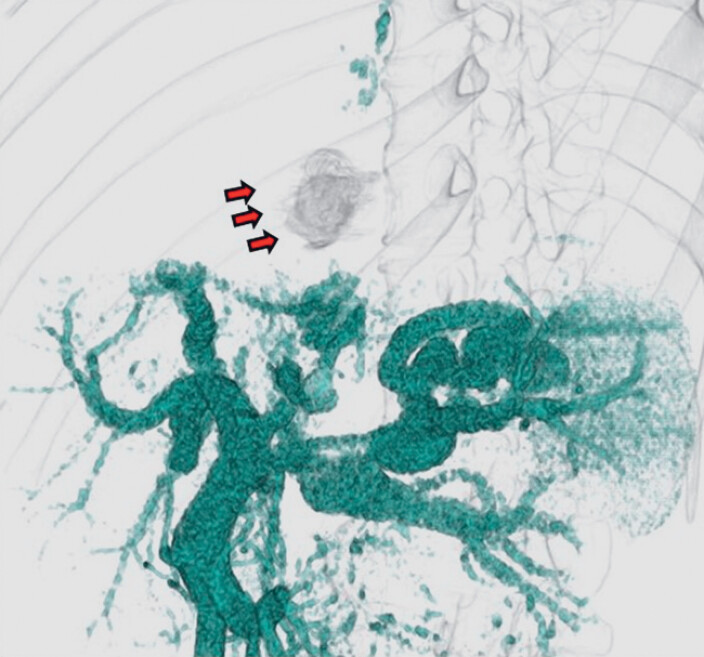
3D computed tomography image 1 week after the procedure showing the coils that had been placed (red arrows) and no further evidence of the varices.

EUS-guided vascular intervention for esophageal variceal bleeding has been previously reported 5 ; however, our new treatment, coiling and sclerotherapy for esophageal varices, has an effect that combines EVL (local blood flow blocking) and EIS (blood flow control including the blood supply route). It is believed this treatment will contribute greatly, even for thick and intractable esophageal varices.

Endoscopy_UCTN_Code_TTT_1AS_2AL

## Notes

Endoscopy E-Videos https://eref.thieme.de/e-videos
*E-Videos* is an open access online section of the journal *Endoscopy* , reporting on interesting cases and new techniques in gastroenterological endoscopy. All papers include a high-quality video and are published with a Creative Commons CC-BY license. Endoscopy E-Videos qualify for HINARI discounts and waivers and eligibility is automatically checked during the submission process. We grant 100% waivers to articles whose corresponding authors are based in Group A countries and 50% waivers to those who are based in Group B countries as classified by Research4Life (see: https://www.research4life.org/access/eligibility/ ). This section has its own submission website at https://mc.manuscriptcentral.com/e-videos .
